# Supplementary figures and images for: Metagenome analysis of viruses associated with Anopheles mosquitoes from Ramu Upazila, Cox’s Bazar District, Bangladesh
Source: PeerJ. 2025 Mar 31;13:e19180. doi: 10.7717/peerj.19180 (PMC11967434; doi:10.7717/peerj.19180)

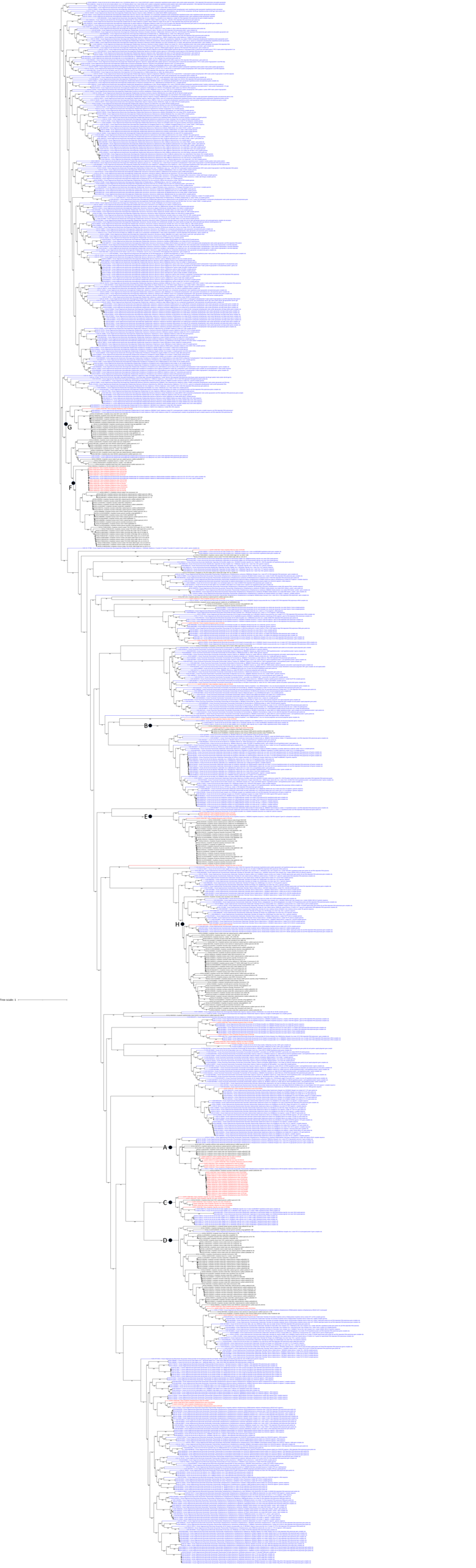

Supplement: Supplemental Information 2 — Nodal support values are the result of 2,000 rapid bootstrap approximations. Major clades containing viral scaffolds described here with evidence of associated endogenous viral elements (EVEs) are labeled A – H. Note that some NCBI viral and Anopheles genome sequences may be represented multiple times in the tree due to non-overlapping (or not completely overlapping) BLASTn hits to the same reference returned by multiple queries during the homology search. [file peerj-13-19180-s002.pdf]
